# Resilience, science, technology, engineering, and mathematics (STEM), and anger: A linguistic inquiry into the psychological processes associated with resilience in secondary school STEM learning

**DOI:** 10.1111/bjep.12496

**Published:** 2022-03-19

**Authors:** Sophie S. Hall, Ross Morrison McGill, Steven Puttick, John Maltby

**Affiliations:** ^1^ College of Life Sciences University of Leicester UK; ^2^ University of Nottingham UK; ^3^ Faculty of Education University of Cambridge UK; ^4^ Department of Education University of Oxford UK

**Keywords:** STEM, resilience, recovery, anger, psychological processes

## Abstract

**Aim:**

To examine resilience in Science, Technology, Engineering, and Mathematics (STEM) learning within an ecological model, identifying the psychological processes associated with resilient, and non‐resilient learning to develop a framework for promoting STEM resilience.

**Sample and method:**

From a sample of secondary‐school students (*n* = 4,936), 1,577 students who found their STEM lesson difficult were identified. Students were assessed on three resilience capabilities and asked to write a commentary on how they responded to the lesson.

**Results:**

Factor analysis revealed that resilience in STEM learning could be positioned within the ecological systems model, with students’ resilience being comprised of three capabilities; the ability to quickly and easily recover (Recovery), remain focussed on goals (Ecological), and naturally adjust (Adaptive capacity). Using a linguistic analysis programme, we identified the prevalence of words within the student commentaries which related to seven psychological processes. Greater ability to recover was negatively related to negative emotional processes. To increase the specificity of this relationship, we identified high and low resilient students and compared their commentaries. Low resilient students used significantly more anger words. Qualitative analysis revealed interpersonal sources of anger (anger at teacher due to lack of support) and intrapersonal sources of anger (including rumination, expression and control, and seeking distraction).

**Conclusions:**

Anger is a key process that distinguishes students who struggle to recover from a difficult STEM lesson. An ecological systems model may prove useful for understanding STEM resilience and developing intervention pathways. Implications for teacher education include the importance of students’ perceptions of teacher support.

## Background

Increasing dependence on technology and forecasted gaps in the supply of Science, Technology, Engineering, and Mathematics (STEM) expertise and skills has made success and retention in STEM education a global policy concern (National Academies of Sciences, Engineering, & Medicine, 2016; United Nations Educational, Scientific and Cultural Organization, 2020). Students' resilience in STEM learning is a key dimension of their achievement and continuation into post‐compulsory STEM. Existing accounts suggest that many students lack the resilience needed to continue STEM studies (Egenrieder, 2010). This is important because of the anticipated increasing demand for STEM skills (Archer, Dawson, DeWitt, Seakins, & Wong, 2015; Christodoulou, 2017) and their role in the economy (Economic Modeling Systems International, 2018; National Audit Office, 2018).

Resilience is needed to overcome the many inherent challenges in these subjects (Haynes, 2008; HM Government, 2017; Ismail, 2018; Millar, 1991), including dealing with the complexity of new and abstract concepts. The effects of these challenges are then confounded by the personal approaches individuals bring to STEM subjects. For example, many students lack the resilience to foster interest, confidence, curiosity, preparedness to fail, and creativity to tackle these subjects (Egenrieder, 2010; Reighard, Torres‐Crespo, & Vogel, 2016). In Morris's, Owens, Ellenbogen, Erduran, and Dunlosky (2019, p. 2) terms, ‘learning supports are critically important for acquiring STEM knowledge because learning opportunities alone are insufficient, as demonstrated by students who attend science class but fail to learn relevant content’. Investigating individual approaches to STEM learning may prove particularly relevant to understanding the STEM gender gap. Despite a number of initiatives, fewer females choose to study STEM post‐16, and there is a lack of females working in the STEM industry (Codiroli Mcmaster, 2017; Smith, 2011). Building resilience skills for STEM across secondary school age will help prepare students for the challenges of future workplaces and job markets, which seem likely to be increasingly unstable and volatile. Therefore, the significance of STEM learning – and resilience in STEM learning in particular – makes the development of more accurate and sophisticated approaches towards understanding resilience in STEM learning an important task for research.

Resilience has typically focused on the development of an individual's strengths, leading to reduced vulnerability to adversity (Edwards, Lunt, & Stamou, 2010), and the term is increasingly being adopted into schools' everyday language (Brown & Dixon, 2020). However, current thinking on resilience is ambiguous and inexact in terms of how it might be applied to STEM learning. In psychology, resilience is often defined *post‐hoc* as any positive factor that leads to a desirable outcome (Maltby, Day, & Hall, 2015), which has generated numerous attempts to define it systematically (Hall, 2010). Researchers currently suggest 15 measures of resilience are suitable for use among young people, which provide over 30 different resilience variables (Constantine & Benard, 2001; Hall, 2010; Jew, Green, & Kroger, 1999; LeBuffe & Naglieri, 1999; LeBuffe, Shapiro, & Naglieri, 2009; Prince‐Embury & Steer, 2010; Rutter, 1985). As such, there are a wide range of resilience variables for researchers and educational practitioners to consider, leading to problems in consistency, accuracy, and theoretical advancements when positioning resilience outcomes within a clear theoretical framework. Fundamentally, this limits the potential for developing strategic approaches to improve resilience in STEM learning.

In response to concern over inconsistencies and disparities in the psychological frameworks for considering and measuring trait resilience (Windle, Bennett, & Noyes, 2011), Maltby et al. (2015) consolidated the most established approaches through a series of factor analyses. The five most well‐cited assessments of resilience, including the Ego Resilience Scale (Block & Kremen, 1996), the Hardiness Scale (Bartone, Ursano, Wright, & Ingraham, 1989), the Psychological Resilience Scale (Wagnild & Young, 1993), the Connor‐Davidson Resilience Scale (Connor & Davidson, 2003), and the Brief Resilience Scale (Smith et al., 2008), covering nine aspects of resilience were included in the analysis. What emerged was that three main resilience capabilities underpin the existing popular assessments of resilience (Maltby et al., 2015). These capabilities can be positioned within the ecological systems, or ecological resilience model originally proposed by Holling (1973, 1996), and therefore suggest that these capabilities are derived from basic natural mechanisms. The capabilities are: *Engineering* (capability to quickly and easily recover), *Ecological* (capability to keep‐going and focussed on key goals), and *Adaptive Capacity* (preference for new and different processes, so naturally adjusting to disturbances; Maltby, Day, Flowe, Day, Flowe, Vostanis, & Chivers, 2019; Maltby, Day, Hall, & Chivers, 2019). In this study, we apply these concepts of resilience to the study of STEM; therefore, using identical terminologies to the ecological model may lead to confusion since Engineering may refer to both a resilience capability and a STEM subject. We, therefore, use ‘Recovery’ to replace 'Engineering resilience' throughout this manuscript.

The ecological resilience model provides a comprehensive framework for assessing resilience capabilities, encompassing and consolidating a range of existing approaches. The concepts of resilience, which were included in the factor analysis, have previously been successfully applied to account for students’ individual responses to dealing with STEM challenges, including ego‐resilience (Donolato, Toffalini, Giofrè, Caviola, & Mammarella, 2020) and hardiness (Daneshamooz & Alamolhodaei, 2012). As such, there is considerable merit in applying the ecological systems model to understand resilience specifically in STEM learning.

It is important that this development work is conducted specifically to a STEM learning context, since students may show resilience in some subjects (e.g., English), but not necessarily others (e.g., Maths). Indeed, research shows that children and young people show resilience to some life stressors but not others (Wright & Masten, 2015). Therefore, applying assessments of general trait resilience, or even general academic resilience, may not provide accurate assessments of resilience in terms of STEM learning. More recent development of the ecological model has shown that with minor modifications, this model of resilience can account for resilience in specific life domains, including education engagement, independent of general trait resilience (Maltby, Day, Flowe, et al., 2019). Specifically, ecological resilience (keeping focussed on goals) is related to higher levels of emotional engagement (e.g., feelings of belonging) and cognitive engagement (e.g., engaging in problem‐solving strategies). In contrast, adaptive capacity (preference for new things) is related to emotional engagement in education (Maltby, Day, Flowe, et al., 2019). Therefore, there is an evident pedigree for translating the ecological model of resilience into a domain‐specific assessment of STEM resilience for secondary school students.

To build a model of resilience in STEM learning that has practical significance, it is essential that we identify the position of students in terms of their resilience capabilities and identify how these relate to broad psychological processes. Identifying how resilience maps onto wider psychological processes would be important for indicating what psychological processes may accompany 'resilient' responses when facing difficulties in STEM learning. Identifying psychological processes is essential for finding ways to promote resilience when needed and address issues of risk around learning when it occurs. The current literature identifies a number of possible psychological areas that are associated with resilience that would map onto STEM learning, including positive expressions of key personality traits, such as conscientiousness and agreeableness, that lead to a specific resilient response to difficult life events (Asendorpf & van Aken, 1999; Robins, John, Caspi, Moffitt, & Stouthamer‐Loeber, 1996), positive expressions of *cognitive* processes, such as using information, planning, and dealing with experiential demands to effectively deal with stressful situations (Cicchetti & Curtis, 2006; Parsons, Kruijt, & Fox, 2016), and positive *emotional* processes that encourage positive growth and the use emotions to deal with adverse situations (Klimoski, 2016; Troy & Mauss, 2011). Therefore, understanding how resilience around STEM learning maps onto psychological processes needs to encompass various psychological factors.

### Study aims

In this paper, we apply the ecological model of resilience to assess aspects of resilience in STEM learning and identify the accompanying psychological processes associated with students’ responses to difficulties in STEM learning. To this end, two aims underpinned the study. The first aim was to develop a psychometric assessment of three core resilience capabilities (Recovery, Ecological resilience [keep focussed on goals], and Adaptive capacity) in the context of experiencing difficulties in STEM learning. This aim led to the first study hypothesis:

- Hypothesis 1: In line with the ecological model of resilience (Holling, 1973; Maltby et al., 2015), we expect to find evidence for three core resilience capabilities in STEM learning. These three resilience capabilities are: the ability to recover (Recovery), the ability to keep focussed on goals (Ecological resilience), and the preference for new things, so able to naturally adjust (Adaptive capacity).

The second aim was to examine how scores on each of the three resilience capabilities are related to self‐described psychological processes around difficulties in STEM learning. We intended to use quantitative and qualitative analyses of these self‐described psychological processes to identify explanations of how resilience informs difficulties in STEM learning. This aim led to two further study hypotheses:

- Hypothesis 2: Based on existing literature suggesting a range of psychological correlates of resilience (Asendorpf & van Aken, 1999; Klimoski, 2016; Parsons et al., 2016), we expect resilience will correlate significantly with self‐described psychological processes around difficulties in STEM learning.
- Hypothesis 3: Comparing the psychological processes described by ‘high’ and ‘low’ resilient students will facilitate specification of the psychological processes associated with resilient and non‐resilient learning in STEM.

## Method

### Participants

Data were collected in nine UK secondary schools from students after a STEM lesson (*n* = 4,936) between September 2019 and January 2020. Ages ranged from 11 to 16 years (UK school years 7–11 compulsory secondary education), 48.5% reported being male. The majority of the sample identified as being ‘White’ (84.5%), followed by ‘Asian’ (6.1%), ‘Mixed’ (2.6%), and ‘Black’ (1.7%); 4.9% of the sample selected ‘Other’ as their ethnic group.

### Measures

Students completed three measures via an online survey (Jisc) administered by the teacher at the end of a STEM lesson. The content of the STEM lesson was not standardized in any way in order to assess students' responses to everyday challenges that they may encounter. The lessons were developed by the teacher, as part of standard planning, to be appropriately pitched for the age and ability level of the class and the research task formed part of the normal lesson. Students were asked by their teachers to complete the survey, but it was made clear that they did not have to participate. The survey comprised three parts. All survey items required a response (it was not possible to skip certain questions or sections), with the exception of the qualitative open‐ended questions (part three).

In part one, students were asked to rate the difficulty of the lesson; ‘How difficult did you find this lesson?’ Responses were scored on a 5‐point scale; 1 = *Not at all difficult*, 2 = *A little difficult*, 3 = *Somewhat difficult*, 4 = *Quite a lot difficult,* 5 = *Extremely difficult*.

In part two, resilience capabilities were assessed by total scores on an adapted version of the 12‐item ecological resilience model, designed to assess naturally occurring resilience traits (Maltby et al., 2015). Small adjustments were made to the items comprising the school‐domain version of the ecological resilience model (Maltby, Day, Flowe, et al., 2019) to develop an assessment of resilience for secondary school learning. For example, ‘I quickly get back to my normal self at school following problems at school’ was changed to ‘It will not take me long to feel ok about myself after the lesson’. The revised scale was developed through an iterative review and piloting process with our Expert Group. Our Expert Group comprised secondary students (*n = *5; 10–16 years); educational psychologists (*n* = 2); and secondary school teachers (*n* = 3). Items were scored on a 5‐point scale from 1 = *Strongly Disagree* to 5 = *Strongly Agree*, with two items being reverse scored.

In part three, to gain insight into the broad psychological processes that accompanied resilience when faced with a difficult STEM lesson, we adopted a qualitative approach. We explored students' written reflections around their STEM lesson so as to understand how they interpret and create meaning around their experience of the STEM lesson (Connelly & Clanndin, 2000). Through a process of linguistic inquiry, we allowed a wide range of important psychological processes to naturally emerge using a method that has been advocated for its validity for exploring mechanisms of action (Holmes et al., 2018).

To produce these responses within a psychological context, students were asked to briefly write a response (student commentaries) to three open‐ended questions based on the well‐recognized triad of affect, cognition, and behaviour found across a series of individual and social psychology theories (Ellis, 1994; Rote & Smettana, 2011; Wilt & Revelle, 2015):

- What did you *feel* when you found something difficult in the lesson?
- What did you *think* about when you found something difficult in the lesson?
- What did you *do* when you found something difficult in the lesson?

### Analysis

#### Missing data

Responses to the survey items were required in order to proceed to the open‐ended items, partly completed or unfinished surveys were not processed by the survey software, and as such there were no missing data.

#### Excluded data

**Figure 1:**
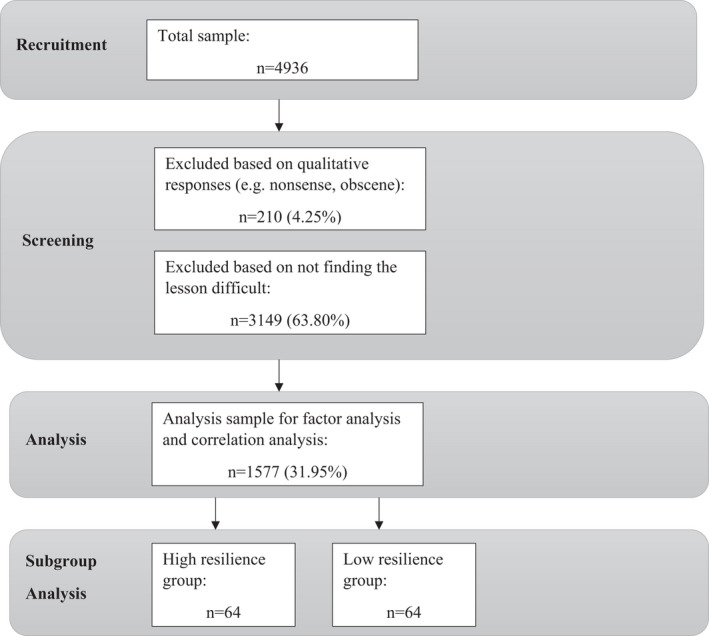
Recruitment and analysis pathway.

Based on theoretical reasons, we excluded responses based on two criteria, and therefore in line with best practice guidelines, we provide the study flow and percentage of excluded data in Figure 1 (APA, 2010; Nicholson, Deboeck, & Howard, 2017). The responses that met these criteria were removed from the final analysis sample. The first criterion was to screen responses the open‐ended items for uncodable data. From the original 4,936 responses, 210 responses were removed based on their commentaries not being suitable for analysis (e.g., using made up words, using emoji's only, or writing obscenities). This was done as we could not be sure the responses given were accurate, and were indicative that the student did not take the survey seriously, thereby leaving a question over the validity of their responses. The remaining 4,726 data sets were assessed against the second criteria, which was students’ perceptions of lesson difficulty. In order to assess the presence of resilience in the STEM classroom, it was important to identify whether students actually found the lesson difficult, and therefore would need to use their resilience in order to keep going with their learning and to be able to respond to the open‐ended questions accurately. Analysis of students' responses to the question pertaining to perceived lesson difficulty showed that 3,149 indicated that they did not find the lesson at least 'somewhat difficult' and these were removed.

#### Analysis sample

The total final sample comprised students who gave coherent responses to the open‐ended questions and reported finding the lesson at least 'somewhat difficult', giving a total analysis sample of *n* = 1,577. Demographic characteristics between the excluded samples were compared using difference tests as recommended in the literature (Nicholson et al., 2017) in the form of Chi‐square tests given the categorical nature of the data. Standardized residuals and adjusted standardized residuals were examined to a cut‐off point of >2 to identify where differences between the excluded group and analysis group were greatest (Sharpe, 2015).

#### Hypothesis 1: There will be evidence for three resilience capabilities, reflected in the ecological resilience model (recovery, ecological resilience, and adaptive capacity), around difficulties in STEM learning

To assess the structural validity of the revised STEM resilience assessment, we examined the structure of the 12‐item ecological resilience measure using Factor Analysis to confirm the three‐factor model as suggested by the ecological model of resilience. We assessed the acceptability of the three‐factor model in two ways, using Confirmatory Factor Analysis as we had a proposed structure. The first way was to explore how well data fitted the three‐factor model using standard goodness‐of‐fit indexes (Hu & Bentler, 1999; Kline, 2005): the chi‐square and degrees of freedom, comparative fit index (CFI), non‐normed fit index (NNFI), root mean square error of approximation (RMSEA), and standardized root mean square residual (SRMR). Whether data fits the proposed model at an ‘acceptable’ level is indicated by a CFI and NNFI greater than .90, and an RMSEA and SRMR of less than .08 (Browne & Cudeck, 1993; Hu & Bentler, 1999; Tabachnick & Fidell, 2001). The second way was to demonstrate the incremental value of proposed model over other theoretically relevant models (Barrett, 2007). Therefore, we compared the three‐factor model for goodness‐of‐fit to a unidimensional model proposing that all 12 items could load on one factor reflecting an underlying latent factor of trait resilience, as opposed to three distinct capabilities. The criteria for showing improved goodness‐to‐fit of the model was ΔCFI > .01 (Chen, 2007). For the proposed three‐factor model, we also included four main steps for testing measurement invariance: configural, metric, scalar, and residual (Waman & Reise, 2004). Measurement invariance between constraints was assessed by a criterion of a change in CFI of less than −.01 change and a change in RMSEA of less than .015 change for metric, scalar, or residual invariance (Chen, 2007; Cheung & Rensvold, 2002; Marsh, Hau, & Grayson, 2005).

#### Hypothesis 2: Resilience will correlate significantly with self‐described psychological processes around difficulties in STEM learning

To analyse the 1,577 written responses for psychological content, we used the Linguistic Inquiry and Word Count (LIWC) programme (Pennebaker, Boyd, Jordan, & Blackburn, 2015). LIWC employs natural language processing and content analysis to process and analyse large amounts of natural language data (Lee, Kim, Lim, & Lee, 2015; Tausczik & Pennebaker, 2010). The LIWC programme has a number of dictionary categories that can be extracted from the text. For each response, LIWC calculates the percentage of total words that match each of the dictionary categories (Tausczik & Pennebaker, 2010). The language categories have been associated with hundreds of psychological terms, but have been grouped to represent broad psychological processes frequently mentioned across the literature (Tausczik & Pennebaker, 2010), including Biological, Drives Perceptual, Positive emotion, Negative emotion, Cognitive, and Social processes. These broad psychological processes can be broken down into their constituent terms (Table 1), for example, Negative emotion comprises three sub‐categories including anger, anxiety, and sadness. The LIWC has been well validated to assess psychological word content within written texts (Schultheiss, 2013).

**Table 1:** Seven broad psychological processes identified by LIWC and examples of their constituent terms

Broad psychological processes	Brief definition	Sub‐categories	Examples
Biological	Process related to ones’ body, digestion, and health	Body, health/illness, sexuality, ingesting	Hands, flu, eat
Drives	Processes related to an individual’s motivations	Affiliation, Achievement, Power, Reward focus, Risk focus	Prize, superior, success
Perceptual	Processes related to seeing and hearing	Seeing, Hearing, Feeling	See, touch, listen
Positive emotion	Processes related to positive affect, being happy	n/a	Happy, love, kind, good
Negative emotion	Processes related to negative affect	Anxiety, Anger, Sadness	Hate, cry, worry, fear, annoyed
Cognitive	Processes related to thoughts or beliefs	Insight, Cause, Discrepancies, Tentativeness, Certainty, Differentiation	Think, know, always, never, maybe, should, could
Social	Processes related to friends, family, social support	Family, Friends, Male referents, Female Referents	Talk, friend, us

We used LIWC to explore the relationship between resilience and psychological processes at two levels. The first was at a broad level, assessing how the three resilience capabilities related to each of the seven psychological processes, to address Hypothesis 2. In terms of assessing the magnitude of the correlations between the measures, we report statistical significance and effect size as frame of reference. We use *r* ≥ .37 as representing a large effect size, .24 ≤ *r* < .37 as representing a moderate effect size, and .1 ≤ *r* < .24 as representing a small effect size (McGrath & Meyer, 2006), with a moderate effect size deemed to be the minimum at which the findings can be considered of practical significance (Cohen, 1992). This criterion differs from the well‐cited effect size of .1 = small to .5 = large, as Cohen based the comparisons with the *d* effect size criteria using a bi‐serial correlation, whilst comparison with the *d* effect size for Pearson product moment correlation coefficients should be based on point bi‐serial correlation (McGrath & Meyer, 2006). It should be noted that a medium effect size is considered as the point at which something gains practical significance generally and in education research (Fan, 2001).

#### Hypothesis 3: Comparing the psychological processes described by ‘high’ and ‘low’ resilient students will facilitate specification of the psychological processes associated with resilient and non‐resilient learning in STEM

The second way we explored the data was to examine for more specific insights, to address Hypothesis 3. We identified a significant relationship between a resilience capability and a psychological process of a medium effect size. We did this using a two‐staged approach, with each stage taking a more detailed examination of the dataset. To facilitate detailed investigations of a voluminous data set, we compared smaller groups of students based on their scores on the resilience capability in question. To this end, groups were created based on high and low resilience scores. This allowed us to identify specific data sets belonging to high and low responders and distinguish between students who described themselves in more resilient terms and those who did not.

The first stage was to look at the relationship between resilience capability and the psychological process more specifically. Instead of just looking at the psychological process in its umbrella term, we also examined its constituent terms (Table 1) to further specify what psychological process accompany resilience following a difficult STEM lesson. Sample size analysis determined that 64 participants were needed for each group (high and low resilience) to achieve a power of .8, *p* < .05 (two‐tailed), specifying a medium effect size (*d* = .5). Therefore, we selected the top (high) and bottom (low) 64 responses on any resilience scale. Where more than 64 responses could be included (e.g., multiple responses shared the same score), we randomly selected from that larger group using random numbers generated from SPSS so that we created equal numbers of data sets. High and low resilient groups were compared on the extent to which their commentaries contained the psychological process of interest (i.e., those that demonstrated a significant correlation with medium effect size in the total data set), including its constituent terms.

The second stage was to visually explore the student commentaries and conduct thematic analysis (Braun & Clarke, 2006) guided by the question 'how and why is this specific psychological process being presented in the student commentaries?'

## Results

### Sample characteristics

Students who were excluded based on their written commentaries not being suitable for analysis (*n* = 210) significantly differed from the analysis sample in terms of age χ^2^(5) = 13.57, *p* < .02; there were proportionally fewer 11‐year‐olds (*excluded*: 8.1% [*n* = 17]; *analysis*: 15.7% [*n* = 248]) and more 14‐year‐olds (*excluded*: 31.9% [*n* = 67]; *analysis*: 22.7% [*n* = 358]) in the excluded sample. There was a significant difference between the two samples in terms of ethnic group χ^2^(4) = 58.35, *p* < .001. The excluded group comprised fewer students reporting as 'White' (*excluded*: 65.7% (*n* = 138); *analysis*: 84.5% (*n* = 1,330) and more students identifying as ‘Asian’ (*excluded*: 17.1% (*n* = 36); *analysis*: 6.1% (*n* = 96), 'Black' (*excluded*: 4.3% (*n* = 9); *analysis*: 1.7% (*n* = 24), and ‘Other’ (*excluded*: 12.9% (*n* = 27); *analysis*: 4.9% (*n* = 127) as their ethnic category. There was also a significant difference between the two samples based on gender χ^2^(2) = 20.21, *p* < .001, with more students in the excluded sample reporting their gender as ‘Other’ (*excluded*: 12.9% (*n* = 27); *analysis*: 5.4% (*n* = 85). Although not the purpose of this research, this highlights that future efforts should be made to encourage engagement with this type of research from non‐White 14‐year‐old students.

Students who were excluded based on not finding the lesson difficult (*n* = 3,149) significantly differed from the analysis sample in terms of age χ^2^(5) = 82.68, *p* < .001, with the excluded sample containing proportionately more 11‐ and 12‐year‐olds (*excluded*: 11‐years: 21.2% [*n* = 669]; 12‐years: 27.5% [*n* = 865]; *analysis*: 11‐years: 15.7% [*n* = 248]; 12‐years 22.4% [*n* = 354]) than the analysis sample, and fewer 14‐ and 15‐year‐olds (*excluded*: 14‐years: 15.6% [*n* = 490]; 15‐years: 10.5% [*n* = 330]; *analysis*: 14‐years: 22.7% [*n* = 358]; 15‐years: 15.8% [*n* = 249]). There was a significant difference between the two samples in terms of gender χ^2^(2) = 6.35, *p* = .04, but the comparison of the residuals did not meet the criteria for further reporting. There was no significant difference between the groups based on ethnic group membership χ^2^(4) = 4.65, *p* = .32. These results suggest that students in earlier secondary school education are less challenged by their STEM lessons than older students, who are likely to be focussing on preparation for formal exams.

### Hypothesis 1: There will be evidence for three resilience capabilities, reflected in the ecological resilience model (recovery, ecological resilience, and adaptive capacity), around difficulties in STEM learning

To test the prediction that student’s resilience in their approach to STEM learning could be assessed in terms of the three main resilience capabilities proposed in the ecological systems model of resilience, confirmatory factor analysis was computed.

#### Confirmatory factor analysis (*n* = 1,577)

The goodness‐of‐fit statistics for the three proposed factor models are presented in Table 2.

**Table 2:** Fit statistics to assess model fit across the proposed models

	χ^2^	*df*	*p* =<	CFI	NNFI	RMSEA	SRMR
Three‐factor	653.998	61	.001	.937	.919	.087	.080
Unidimensional	2,479.580	54	.001	.748	.691	.169	.126

The three‐factor model met the acceptable fit criteria across the indexes of CFI, NNFI, RMSEA, and SRMR, whereas the unidimensional model failed to meet these criteria. The three‐factor model was evaluated to assess for measurement invariance across both gender (identified as 'male', 'female', or 'other') and age (10–13 and 14–16 years) groups. Successively stricter constraints were tested across groups to evaluate configural, metric, scalar, and residual invariance (Widaman & Reise, 2004). Table 3 demonstrates the fit statistics for multi‐group factor confirmatory factor analysis for the bi‐factor model by gender and age. These findings suggest measurement invariance occurred for gender and age up to the residual level.

**Table 3:** Fit statistics for multi‐group factor confirmatory factor analysis for the resilience scale by gender and age

	χ^2^ (*df*)	RMSEA	SRMR	CFI	NNFI	ΔRMSEA	ΔCFI
Gender							
Configural	837.22 (153)	.053	.060	.928	.906		
Metric	874.68 (171)	.051	.062	.926	.914	−0.002	−0.002
Scalar	946.67 (192)	.050	.061	.920	.918	−0.001	−0.006
Residual	1,008.49 (216)	.048	.061	.916	.923	−0.002	−0.004
Age							
Configural	728.98 (102)	.062	.065	.935	.916		
Metric	786.68 (111)	.062	.071	.930	.917	0.000	−0.005
Scalar	808.77 (121)	.060	.071	.929	.923	−0.002	−0.001
Residual	841.49 (133)	.058	.073	.927	.927	−0.002	−0.002

### Hypothesis 2: Resilience will correlate significantly with self‐described psychological processes around difficulties in STEM learning

To test the prediction that student commentaries would contain key psychological processes associated with resilience capabilities, Pearson’s correlations were computed.

Table 4 provides Pearson’s correlations between the three resilience capabilities and the seven psychological processes at a general level. In terms of correlations between resilience and psychological processes that reach a medium effect size (*r* ≥ .24), the capability to recover shares a significant negative correlation with the use of negative emotional words. In terms of the specific components of negative emotional words (anxiety, anger, sadness), none of the correlations had a magnitude that reached a medium effect size: anxiety, *r* = −.105, *p* < .001; anger, *r* = −.119, *p* < .001; sadness, *r* = −.162, *p* < .001. This suggests that correlational analyses of these components do not increase the specificity of the possible relationship between the capability to recover and negative emotion.

**Table 4:** Pearson’s correlations for the total sample (*n* = 1,577) between student resilience capabilities and seven psychological processes extracted from LIWC

	1	2	3	4	5	6	7	8	9	10
Resilience capabilities										
Recovery	1	.224**	.102**	.118**	−.243**	.020	.115**	.009	−.015	.002
Ecological resilience		1	.628**	.069**	−.217**	.002	.023	.071**	−.078**	.041
Adaptive capacity			1	.086**	−.167**	.007	.048	.048	−.083**	.064**
LIWC psychological processes										
Emotion (positive)				1	−.206**	−.047	−.008	.056*	−.010	.121**
Emotion (negative)					1	−.035	−.077**	−.107**	.034	−.011
Social processes						1	−.167**	−.202**	−.113**	.315**
Cognitive processes							1	−.006	−.096**	−.184**
Perceptual processes								1	.072**	−.095**
Biological processes									1	−.084**
Drives										1

### Hypothesis 3: Comparing the psychological processes described by ‘high’ and ‘low’ resilient students will facilitate specification of the psychological processes associated with resilient and non‐resilient learning in STEM

To test the prediction that comparing high and low resilient learners would enable further specification of the processes associated with resilient and non‐resilient learning, we compared students who scored high and low on the resilience scales. More specifically, to increase the specificity of the relationship between the ability to recover and negative emotion words, we identified the individual resilience scores and comments made by students who scored high and low on this resilience sub‐scale. For the 'high' resilient group, 164 responses obtained the highest possible score on the 'recovery' scale; therefore, we randomly selected 64 individuals to form the 'resilient' group. Low responses ranged from 3 to 5, with 30 individuals scoring 5, leading to 79 responses falling into this range. Therefore, we selected a random subsample from those scoring 5, to create a sample of *n* = 64. There were significantly more males in the high resilient group (70.3%) compared to the low resilient group (25%) *χ* ^2^(1) = 25.38, *p* < .001 (see Supplementary Table 1 for resilience scores by age group). No significant differences were observed between the two samples in terms of age *χ^2^* (6) = 5.36, *p* = .49 or ethnic group χ^2^(3) = 1.77, *p* = .62. Table 5 shows the *t*‐test statistics for the high and low 'recovery' groups for negative emotion overall and the constituent terms. Here we find a significant difference between students who self‐reported 'high' and 'low' resilience for negative emotion, to a large effect size. In terms of the constituent terms, it is only for anger we find a significant difference that meets a medium effect size.

**Table 5:** Mean percent data, *t*‐test statistics, and effect sizes (Cohens *d*), comparing high and low resilient students’ use of negative emotion words following a difficult STEM lesson

	Low resilience (*n* = 64)	High resilience (*n* = 64)	*t*	*p*	Effect size
	Mean % (*SD*)	Mean % (*SD*)			
Negative emotion	13.76 (13.23)	4.60 (7.37)	4.85	.001	0.86
Anxiety	3.17 (5.41)	1.58 (4.27)	1.85	.067	0.33
Anger	4.85 (9.55)	0.78 (2.42)	3.31	.001	0.58
Sadness	3.42 (5.44)	1.12 (5.12)	2.46	.015	0.44

To illustrate the presence of these anger terms and further elucidate the mechanisms driving anger, we qualitatively explored the student commentaries. Two themes emerged from this process (see Table 6), representing the two key sources of anger. The first source (theme 1) was related to a perceived lack of teacher support, which we termed interpersonal sources of anger. Students reported feeling angry that their teacher did not provide them with the support they needed. Students also felt that their teacher did not care about them and that asking for help would lead to a negative reaction. The second source (theme 2) reflected a range of factors that have been associated with anger in the literatures, including rumination (Sukhodolsky, Golub, & Cromwell, 2001), anger‐frustration (Fox & Spector, 1999), anger expression and control (van Elderen, Maes, Komproe, & van der Kamp, 1997), and seeking distraction (Kubiak, Wiedig‐Allison, Zgoriecki, & Weber, 2011). We termed these intrapersonal sources of anger.

**Table 6:** Anger comments contained in the commentaries of low resilient students

*Theme 1: Interpersonal Sources of Anger*	
Teacher support	‘How upset and unimpressed my teacher is’
	‘Nothing because our teacher doesn't help’
	‘Miss is going to have ago at me if I ask’
	‘Hold my hands up for about half an hour for no one to come and help me because the teacher cannot teach/help’
*Theme 2: Intrapersonal Sources*	
Rumination (Sukhodolsky et al., 2001)	‘Really annoyed and worried’
	‘I felt worried’
	‘Like an idiot’
	‘I am so stupid why do I even try I will never get it right’
	‘Sad very sad’
	‘Dreadful’
	‘Depressed’
	‘Upset and stressed and a lot of anxiety’
	‘Like I would fail in life’
Anger‐frustration	‘Angry sad annoyed frustrated’
	‘Anger, frustration and depression’
	‘Tired and annoyed’
Anger Expression & Control (van Elderen et al., 1997)	‘Like throwing fists lad, I was EXTREMELY INFURIATED’
	‘I got angry’
Seeking Distraction (Kubiak et al., 2011)	‘Thought about going home’
	‘I think about random things’
	‘I zone out and daydream….’
	‘I just doodle’

## Discussion

The findings confirmed the three study hypotheses. The first hypothesis was that we would find evidence for three resilience capabilities (Recovery, Ecological resilience, and Adaptive capacity) around difficulties in STEM learning. We were able to show, through confirmatory factor analysis, that a three‐factor model provided the best fit. The second hypothesis was that resilience would significantly correlate with self‐described psychological processes around difficulties in STEM learning. Students’ written descriptions of how they dealt with difficulties in STEM learning contained key psychological processes which significantly correlated with resilience, most notably negative emotional processing was associated with a lower ability to ‘Recover’. Finally, the third hypothesis was that comparing the psychological processes described by ‘high’ and ‘low’ resilient students would facilitate specification of the psychological processes associated with resilient and non‐resilient learning in STEM. By identifying students who scored the highest and the lowest on the Recovery scale, we explored what these students described in relation to negative emotional processing. This enabled us to identify anger as a key process that distinguished students who were able to easily ‘Recover’ (high resilient learners) and those who could not easily ‘Recover’ (low resilient learners).

This study provides the first attempts, known to the authors, to develop a framework of resilience that is specific to STEM learning. Previous research has successfully applied the ecological model of resilience to understanding resilience in specific life‐domains, including work, health, marriage, friendships, and education (Maltby, Day, Flowe, et al., 2019). Here, we demonstrated the utility of applying the ecological model to STEM resilience. Individuals make different assessments of their levels of resilience across different domains (Vanderbilt‐Adriance & Shaw, 2008); therefore, it is important that resilience is assessed in relation to specific contexts in order to develop more sophisticated and useful models of resilience in education. Studies show that resilience is linked to educational engagement within school, university, and alternative education settings (Cotton, Nash, & Kneale, 2017; Zolkoski, Bullock, & Gable, 2016) and more specifically, to persisting in STEM programmes (Bekki, Smith, Bernstein, & Harrison, 2013). This initial work shows that by using a 12‐item scale, we were able to reliably assess (demonstrated through model fit indexes) three resilience capabilities specific to a STEM learning context; these resilience capabilities are well documented in the literatures and have been shown to underpin multiple existing model of general trait resilience (Maltby et al., 2015). This provides a useful model for conceptualizing and assessing resilience in the STEM classroom, promoting practitioner understanding and competence for discussing resilience in this context and for identifying at‐risk students. It is proposed, congruent with Hypothesis 1, that resilience in STEM can be conceptualized in terms of Recovery – the ability to keep going, Ecological resilience – the ability to keep focussed on goals, and Adaptive capacity – the ability to naturally adjust. Furthermore, we developed these concepts into a framework of STEM resilience by identifying the psychological processes which accompany high and low resilience capabilities. This will enable future research to test the importance of specific resilience capabilities in determining learning outcomes and highlight pathways to promote STEM engagement at secondary school level. Here we began to uncover some of these pathways by identifying the salient psychological processes associated with specific resilience capabilities (Hypothesis 2).

Anger emerged as a key psychological process associated with the ability to recover during a difficult STEM lesson, distinguishing students with high and low abilities to recover (Hypothesis 3). Previous research has identified anger as key learning emotion (Pekrun, 2017; Pekrun, Lichtenfeld, Lichtenfeld, Marsh, Murayama, & Goetz, 2017), and it has been acknowledged that students experience frustration when encountering difficult STEM concepts (King, Ritchie, Sandhu, Henderson, & Boland, 2017). Given that our analysis sample comprised students who all reported finding the lesson at least 'somewhat difficult', it is perhaps not surprising that anger was identified in the students’ written commentaries. However, this is the first time that anger has been specifically identified in relation to resilience capabilities. This is important because anger was most evident in the commentaries of low resilient students. This identifies a clear pathway for preventing students from giving up on their STEM learning (i.e., by reducing feelings of anger and frustration).

Qualitative analysis revealed that anger appeared to have two sources. The first, termed 'interpersonal sources of anger', reflected that students felt annoyed by the lack of teacher support. This is consistent with the general educational literatures which identify the importance of teacher–student relatedness in determining academic outcomes (León & Liew, 2017; Quin, 2017). More specifically, previous research suggests teacher support is an importance factor in persisting with STEM (Fredricks, Hofkens, Wang, Mortenson, & Scott, 2018; Simpkins, Liu, Hsieh, & Estrella, 2020; Trigueros et al., 2019). Increased teacher support is associated with a better self‐concept (Simpkins et al., 2020), attitude (Vennix, den Brok, & Taconis, 2018), and motivation (Jungert, Levine, & Koestner, 2020) to learn about STEM. In particular, teacher support has been shown to be important in determining STEM engagement from female students (Buday, Stake, & Peterson, 2012; Rice, Barth, Guadagno, Smith, & McCallum, 2013; Wang & Degol, 2013), and it is evident that the low resilient group in this study comprised more female than male students. Nonetheless, based on the current findings, it is unclear whether teacher behaviour is a contributing factor in determining anger, indeed literatures indicate that the teacher can influence emotions experienced in the STEM classroom (Trigueros et al., 2019), or whether expressions of anger are characteristic of narrowing and 'framing' whereby the teacher becomes the enemy (Beck & Deffenbacher, 2000) during a difficult lesson.

The second source, termed 'intrapersonal sources of anger', reflected a range of variables associated with anger, including rumination, anger‐frustration, anger expression and control, and seeking distraction. 'Recovery' reflects an individual's ability to quickly and easily return to a stable state, and it is clear that these factors would inhibit this.

Anger rumination is characterized by repetitive thinking about being angry or an anger‐inducing situation (Sukhodolsky et al., 2001). Anger rumination was evidenced in the commentaries of low resilient students, who referred to feeling worried and over‐extrapolated the experience to believing they were a failure and would never get things right. These persistent thoughts can result in task irrelevant thinking (Pekrun et al., 2004) and impede enjoyment and achievement in the classroom (Pekrun, Murayama, Marsh, Goetz, & Frenzel, 2019) as well as having a negative impact on self‐esteem (Turner & White, 2015; Waschull & Kernis, 1996).

Anger from frustration could be inferred from the comments, whereby a students' response was to write words like 'tired and annoyed', and 'anger, frustration and depression'. As discussed earlier, given that the students reported finding the lesson difficult, it is perhaps logical to assume this causes some form of frustration. However, these feelings of frustration were most evident in students reporting the lowest resilience. As such, feeling frustrated may be a unique feature of low resilience during a challenging lesson, not just dealing with a challenging lesson per se.

There was some evidence to suggest that students struggled to inwardly control their anger, resulting in an outward anger expression (van Elderen et al., 1997). Research suggests that teacher support is important in controlling expressions of anger (Spaulding, 2005), and theme 1 reflects that low resilient students feel unsupported by their teachers. Therefore, not only may improving student perceptions of teacher support help promote STEM engagement, but it also may help the teacher control the classroom and promote a positive classroom climate (Rucinski, Brown, & Downer, 2018; Walker & Graham, 2019). However, a distinction should be made between providing support, and appearing over controlling, since directly controlling teacher behaviours are associated with increased student anger (Assor, Kaplan, Kanat‐Maymon, & Roth, 2005).

Finally, low resilient students appeared to seek distraction from the event. Seeking distraction is an anger regulation strategy often learnt during early childhood, which enables feelings of anger to dissipate (Cole et al., 2011; Drake & Winner, 2013). However, whilst distraction techniques may be effective when waiting for something desirable, they are less effective when a child has to solve a problem (Sethi, Mischel, Aber, Shoda, & Rodriguez, 2000; Tan, Armstrong, & Cole, 2013). As such, caution should be taken when applying to the development of classroom‐based anger regulation strategies.

No salient associations were identified between the two other resilience capabilities (ecological resilience and adaptive capacity) and psychological processes. However, the psychological processes investigated here were constrained to those captured within the LIWC dictionaries, and this represents the first limitation of the study. Whilst LIWC has been developed to provide an objective, comprehensive assessment of linguistic content (Tausczik & Pennebaker, 2010), a more subjective thematic qualitative analysis may reveal other links to psychological processes. Nonetheless, the approach taken here enabled us to combine objective, quantitative comparisons with more detailed qualitative investigations to avoid biasing interpretation of the data.

The only demographic factor that distinguished the high and low resilient group was gender; with the low resilient group comprising more females than the high resilient group. This is congruent with the STEM gender gap, in that females are less likely to continue with STEM learning than their male counterparts (Raabe, Boda, & Stadtfeld, 2019; Wang & Degol, 2017). The findings reported here suggest that a lack of STEM resilience may partly explain the gender gap as opposed to a lack of STEM ability. Whilst we broadly assessed ethnic identity and age, we did not explore other factors that could prove important, such as socioeconomic status, which represents a second limitation of the study. Indeed, evidence suggests that lower income students often perform poorly and do not persist with STEM subjects (Saw, Chang, & Chan, 2018), and this is at least in part related to their ability to control negative emotions (Rozek, Ramirez, Fine, & Beilock, 2019). A third limitation study is that individual differences reflecting personality dimensions were not directly assessed. Therefore, it is not possible to tell whether individuals prone to anger (i.e., high in trait anger) are more vulnerable to low resilience when encountering STEM challenges or the challenge itself that leads to anger. This represents an important point for future research.

The link between negative emotion and specific resilience capabilities in STEM learning is congruent with the emerging literature that identifies the importance of emotion regulation in STEM learning (Rozek et al., 2019; Sokolowski, Hawes, & Lyons, 2019) this has clear implications for practice. Schools may want to consider implementing strategies to regulate emotion to help students quickly and recover when faced with a difficult topic. Indeed, other research suggests that resilient individuals use strategies to regulate negative emotions, such as engaging in relaxation (e.g., allowing time to interpret and assess problems), exploration (e.g., consider alternatives), and optimistic thinking (e.g., having faith to overcome the challenge; Werner & Smith, 1992). These strategies could be applied to learning in the STEM classroom to encourage secondary school students to persist with their STEM education.

In summary, the current findings suggest that the ecological model of resilience, comprising recovery (quickly and easily return to a stable state), ecological resilience (keep‐going and focussed on goals) and adaptive capacity (preference for new things), can be applied as useful framework to understand and assess resilience in secondary school STEM education. We extend our understanding of the ecological model by identifying important psychological processes associated with low resilience capabilities to begin to position STEM resilience within a framework that has clear practical implications. More specifically, students with a low ability to recover were identified by their use of anger words when describing how they dealt with a difficult lesson.

Our findings add to previous understandings of the importance of teachers' relationships by emphasizing the significance of students' perceptions of their teacher's support. What it means to be seen to offer and be available to provide support to students will vary in different contexts. Our study provides further support for teacher education and continuing professional development to highlight the importance of nurturing classroom environments in which individual students feel that they are supported at the same time as being challenged by the subject matter. Teachers might consider reflecting on the different ways in which support is offered to students and how students feel about these different options. Perhaps most importantly, a point to consider is whether students recognize the different ways teachers are trying to offer support, and if not, identify what changes can be made to make support more evident, more accepted by students, and more effective.

## Conflicts of interest

All authors declare no conflict of interest.

## Author contribution

**Sophie Susannah Hall:** Conceptualization; Data curation; Formal analysis; Funding acquisition; Methodology; Project administration; Writing – original draft; Writing – review & editing. **Ross Morrison McGill:** Resources; Supervision; Validation; Writing – review & editing. **Steven Puttick:** Funding acquisition; Investigation; Supervision; Validation; Writing – review & editing. **John Maltby:** Conceptualization; Data curation; Formal analysis; Funding acquisition; Methodology; Project administration; Resources; Supervision; Writing – review & editing.

## Ethical approval

Ethical approval was received from (blinded for review).

## Supporting information

Table S1 Comparison of Resilience Mean Scores by Gender for each Age group (11‐16 years) (Mean ± Standard Deviation)

## Data Availability

The data that support the findings of this study are available on request from the corresponding author. The data are not publicly available due to privacy or ethical restrictions.
