# Supplementary material for: Resilience, science, technology, engineering, and mathematics (STEM), and anger: A linguistic inquiry into the psychological processes associated with resilience in secondary school STEM learning
Source: Br J Educ Psychol. 2022 Mar 19;92(3):1215–38. doi: 10.1111/bjep.12496 (PMC9545978; doi:10.1111/bjep.12496)
Supplement: Supplementary file 1 — Table S1 Comparison of Resilience Mean Scores by Gender for each Age group (11‐16 years) (Mean ± Standard Deviation) [file BJEP-92-1215-s001.docx]

Supplementary Table 1

*Comparison of Resilience Mean Scores by Gender for each Age group (11-16 years) (Mean ± Standard Deviation)*

|  | 11-years | | |  | 12-years | | |  | 13-years | | |
| --- | --- | --- | --- | --- | --- | --- | --- | --- | --- | --- | --- |
| Scales | Male  (n=149) | Female  (n=100) | t |  | Male  (n=196) | Female  (n=135) | t |  | Male  (n=154) | Female  (n=134) | t |
| Recovery | 10.98±2.49 | 10.24±2.79 | 2.21* |  | 11.43±2.68 | 9.78±2.60 | 5.56** |  | 11.41±9.54 | 9.54±2.95 | 5.44** |
| Ecological | 15.26±2.56 | 15.61±2.90 | -0.98 |  | 15.23±2.86 | 14.29±2.86 | 2.94** |  | 14.89±2.85 | 13.96±2.95 | 2.72** |
| Adaptive | 15.77±3.31 | 15.40±3.17 | 0.90 |  | 14.54±3.44 | 14.02±3.66 | 1.30 |  | 14.02±3.42 | 13.40±3.93 | 1.44 |
|  |  | | |  |  | | |  |  | | |
|  | 14-years | | |  | 15-years | | |  | 16-years | | |
|  | Male  (n=153) | Female  (n=181) | t |  | Male  (n=109) | Female  (n=133) | t |  | Male  (n=22) | Female  (n=26) | t |
| Recovery | 11.28±2.37 | 9.56±2.92 | 5.82** |  | 10.40±2.62 | 9.50±2.96 | 2.47* |  | 10.27±8.84 | 8.85±2.57 | 1.80 |
| Ecological | 15.09±3.05 | 13.97±2.97 | 3.39** |  | 15.16±2.90 | 14.77±2.94 | 1.03 |  | 14.45±3.06 | 13.76±3.39 | 0.73 |
| Adaptive | 14.79±3.54 | 13.66±3.76 | 2.81** |  | 15.31±3.06 | 14.58±3.40 | 1.73 |  | 13.90±3.61 | 14.00±3.48 | -0.09 |

Key: *p < .05; ** p<.01. NB: This table is included for reference only and did not form part of the study aims. This table provides an insight into when resilience capabilities may begin to differ between male and female students in our total analysis sample (*n*=1577). Missing gender data represents respondent indicated ‘other’ gender (*n*=85)
